# Strategies used by two apterous strains of the pea aphid *Acyrthosiphon pisum* for passive dispersal

**DOI:** 10.1242/bio.018903

**Published:** 2016-09-14

**Authors:** Yi Zhang, Xing-Xing Wang, Jing-Yun Zhu, Zhan-Feng Zhang, Hong-Gang Tian, Tong-Xian Liu

**Affiliations:** College of plant protection, State Key Laboratory of Crop Stress Biology for Arid Areas, and Key Laboratory of Integrated Pest Management on the Loess Plateau of Ministry of Agriculture, College of Plant Protection, Northwest A&F University, Yangling, Shaanxi 712100, China

**Keywords:** Apterae, Locomotion, Adaptation, Natural selection, Starvation

## Abstract

Wingless forms of aphids are relatively sedentary, and have a limited ability to migrate or disperse. However, they can drop off hosts or walk away if disturbed, or their food quality or quantity become deteriorated. Earlier, we found that the pea aphid, *Acyrthosiphon pisum* (Harris, 1776), could use differed strategies to escape danger and locate new host plants. To determine the mechanisms behind the different strategies, we undertook a series of studies including the aphids' host location, energy reserves under starvation, glycogenesis, sugar assimilation, olfactory and probing behaviors. We found that in our controlled laboratory conditions, one strain (local laboratory strain) moved longer distances and dispersed wider ranges, and correspondingly these aphids assimilated more sugars, synthesized more glycogen, and moved faster than another strain (collected from Gansu Province, northwestern China). However, the latter strain could locate the host faster, probed leaves more frequently, and identified plant leaves more accurately than the former strain after they were starved. Our results explained how flightless or wingless insects adapt to fit biotic and abiotic challenges in the complex processes of natural selection.

## Abstract (precis)

**Summary:** Two strains of pea aphid (*Acyrthosiphon pisum*) show different dispersal strategies, glycogen level and probing behaviors. One strain [Yangling strain (YL)] moves longer and disperses wider than another strain [Lanzhou (LZ)] based on higher glycogen. LZ strain probed more frequently, and identified plant more accurately than YL strain.

## INTRODUCTION

Many insects show polyphenism for adaptations to environmental conditions, including aphids which have winged and wingless phenotypes as longer-dispersing and shorter-dispersing morphs (Braendle et al., 2006). Density (tactile stimulation) and nutrition (host plant quality) are considered to be the key factors which influence the production of winged or wingless morphs, and increased population density always leads wing formation in many aphid species (Johnson, 1965; Lees, 1967; Sutherland, 1969; Sutherland and Mittler, 1971; Wratten, 1977). Winged individuals (alatae) have functional flight muscles and other characters relating to flight and host-plant location, while wingless individuals (apterae) are mostly sedentary and walk only short distances (Braendle et al., 2006; Brisson, 2010; Irwin et al., 2007); but their dispersal behaviors are also important. For instance, the pea aphid, *Acyrthosiphon pisum* (Harris 1776), could partake in within-ﬁeld dispersal to distant plants (Ben-Ari et al., 2015). Studies on apterae of *Macrosiphum euphorbiae* and *Myzus persicae* showed different dispersal behaviors and moment abilities (Alyokhin and Sewell, 2003). Many apterous aphids could increase their rate of locomotion and decrease their orientation to vertical image after being dislodged by an alarm pheromone (Phelan et al., 1976; Kunert et al., 2010).

Some studies indicate that aphids can detect plant volatiles for host finding (Chapman et al., 1981; Webster et al., 2008; Döring, 2014), while others minimized the role played by plant volatiles (Brady, 1997; Gish and Inbar, 2006; Döring, 2014). For instance, an early study showed that the host location of the apterous *Macrosiphoniella artemisiae* (Boyer de Fonscolombe 1841) was based on visual cues, and host plant odors did not play a major role in host location (Brady, 1997). Some aphids, such as *A. pisum*, also show polymorphism in body colors (Libbrecht et al., 2007; Losey et al., 1997), and differ genetically (Caillaud and Losey, 2010; Moran and Jarvik, 2010); however, the connection between the body color and their dispersal behaviors is still unclear (Caillaud and Losey, 2010; Tabadkani et al., 2013).

Aphids in wild conditions are always facing natural enemies, including mammalian herbivores (Völkl et al., 2007). Some aphids, especially wingless individuals including *A. pisum*, can avoid predators by dropping off the host plant, and this behavior could also be affected by physical and biological environmental conditions (Roitberg and Myers, 1979; Dill et al., 1990; Bailey et al., 1995; Mann et al., 1995; Gish et al., 2010). This escaping response could be triggered by the herbivore's breath or aphid pheromone (Roitberg and Myers, 1979; Dill et al., 1990; Gish et al., 2010).

The dispersal or migration behaviors in *A. pisum* varied among sympatric populations based on their original hosts (Peccoud et al., 2009), and also differed in defensive behaviors (response to aphid alarm pheromone). It is believed that different plants could give different natural selection pressures or nutrition which could affect the behaviors of *A. pisum* (Phelan et al., 1976; Kunert et al., 2010)*.* Studies on another aphid, *Nasonovia ribisnigri* (Mosley 1841), showed that distribution patterns and dispositions of apterous were different in different seasons (Díaz et al., 2012).

During our studies on two strains of apterous *A. pisum*, we observed that the two strains of apterous *A. pisum* exhibited different passive dispersal behaviors, escaping strategies, and host finding and locomotion abilities. We realized that these phenomena may imply that *A. pisum* might have evolved more than one countermeasure for certain threats. Therefore, we designed this study to determine why these two wingless strains of *A. pisum* have different dispersal strategies. We had four objectives in this study: (1) to confirm and quantify the food location behaviors of the two wingless strains after they were starved; (2) to determine the host plant location and locomotion patterns of the two wingless strains; (3) to determine if the amount of energy reserves and related key enzymes of the two wingless strains were related to their dispersal behaviors; and finally, (4) to determine the host searching behaviors using small arenas and Y-tube olfactometers, and to compare their probing behaviors using an electrical penetration graph (EPG) system.

## RESULTS

### Body lengths and weights of the two pea aphid strains

**Fig. 1.**
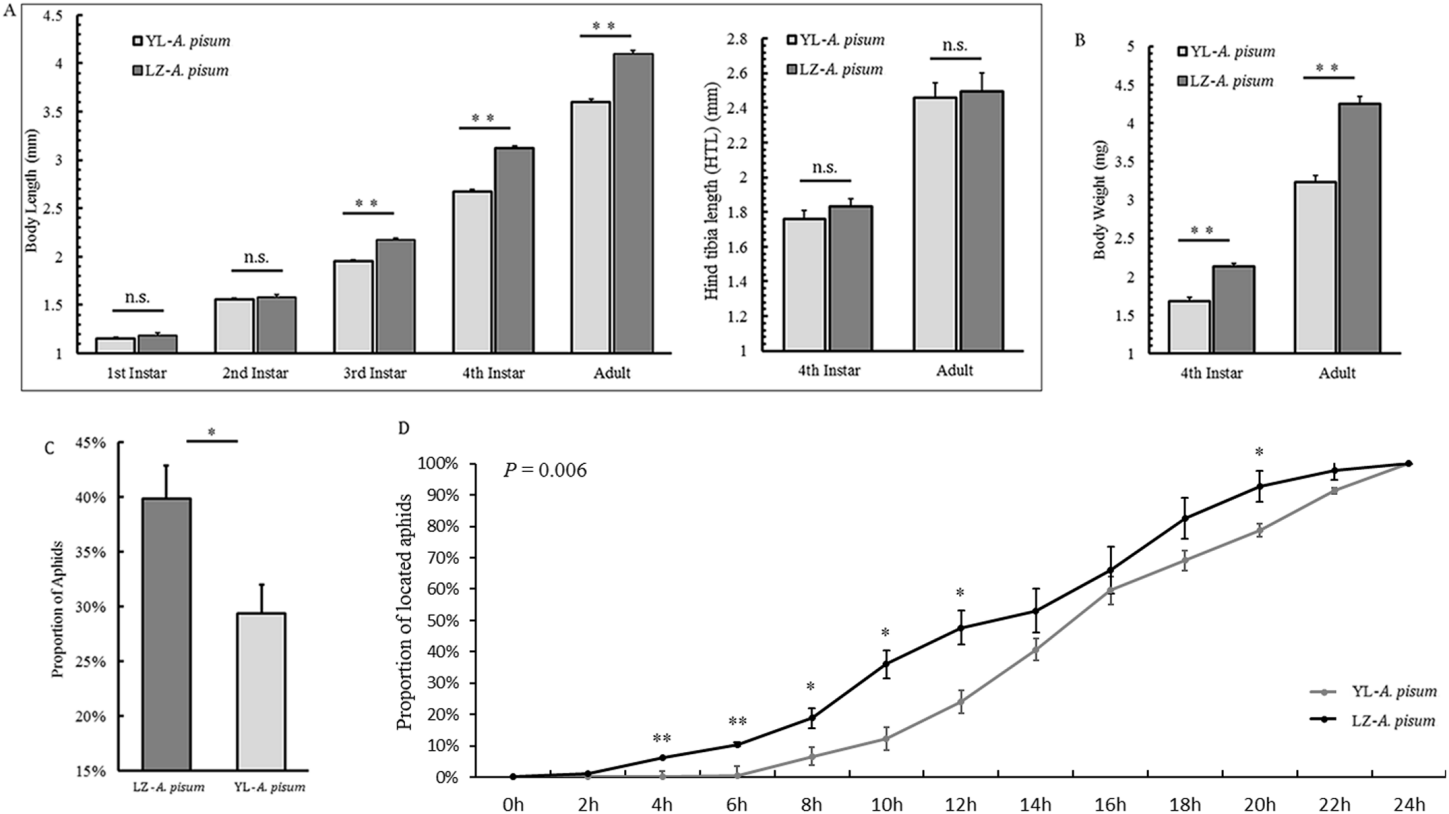
**Biological and host-seeking differences between two selected pea aphids.** Body length, hind tibia length (third pair of legs) (A) and weight (B) differences between two selected host-seeking wingless strains of *Acyrthosiphon pisum*. (C) Proportions of aphids that reached the host leaf disc after 24 h. (D) Percentage increase of aphids that located in every two hours in relation to total numbers of aphids on the host leaf disc in 24 h. Values represent mean±s.e.m. from independent determinations; *P<0.05, **P<0.01 (Student's *t*-test in B and C; parameter estimates of general linear model repeated-measures in D). *P* values in D derived with repeated measures testing.

The Lanzhou (LZ) strain body-length of third, fourth and fifth instar were longer than those of the Yangling (YL) strain [third: *t*=7.516, d.f.=116, *P*<0.001; fourth: *t*=13.59, d.f.=116, *P*<0.001; fifth (adult): *t*=11.25, d.f.=116, *P*<0.001; Fig. 1A], but no significant difference was observed in the first and second instar nymphs between the two strains (first: *t*=1.443, d.f.=116, *P*=0.152; second: *t*=0.963, d.f.=116, *P*=0.337; Fig. 1A), and the tibia length of the third pair of legs showed no difference between two strains [fourth: *t*=1.881, d.f.=98, *P*=0.063; fifth (adult): *t*=0.856, d.f.=98, *P*=0.394; Fig. 1A]. Body weight also had different results between the two strains, the LZ strain in fourth and fifth instar were significant heavier than the YL strain [fourth: *t*=6.604, d.f.=100, *P*<0.001; fifth (adult): *t*=7.549, d.f.=100, *P*<0.001; Fig. 1B].

### Host location

The LZ strain was more effective in plant location than the YL strain, and more aphids in the LZ strain were observed on the host leaves than those in the YL strain (39.8% vs 29.4%) after 24 h (*t*=2.604, d.f.=18, *P*=0.018) (Fig. 1C). The population-increase patterns of the two strains differed, and the first LZ aphid reached the host leaf approximately in 1 h, whereas the first YL strain aphid reached the leaf in approximately 5 h. The multivariate tests of repeated-measures (GLM, General Linear Model) showed significant interactions between time and experimental strains (*P*=0.003); and the tests of between-subject effects (repeated-measures of GLM) showed that the proportions of the locations of the two aphids' strains in 24 h were significantly different (*F*=9.482, d.f.=1, 18, *P*=0.006) (Fig. 1D, Fig. S4).

### Locomotion under starvation

**Fig. 2.**
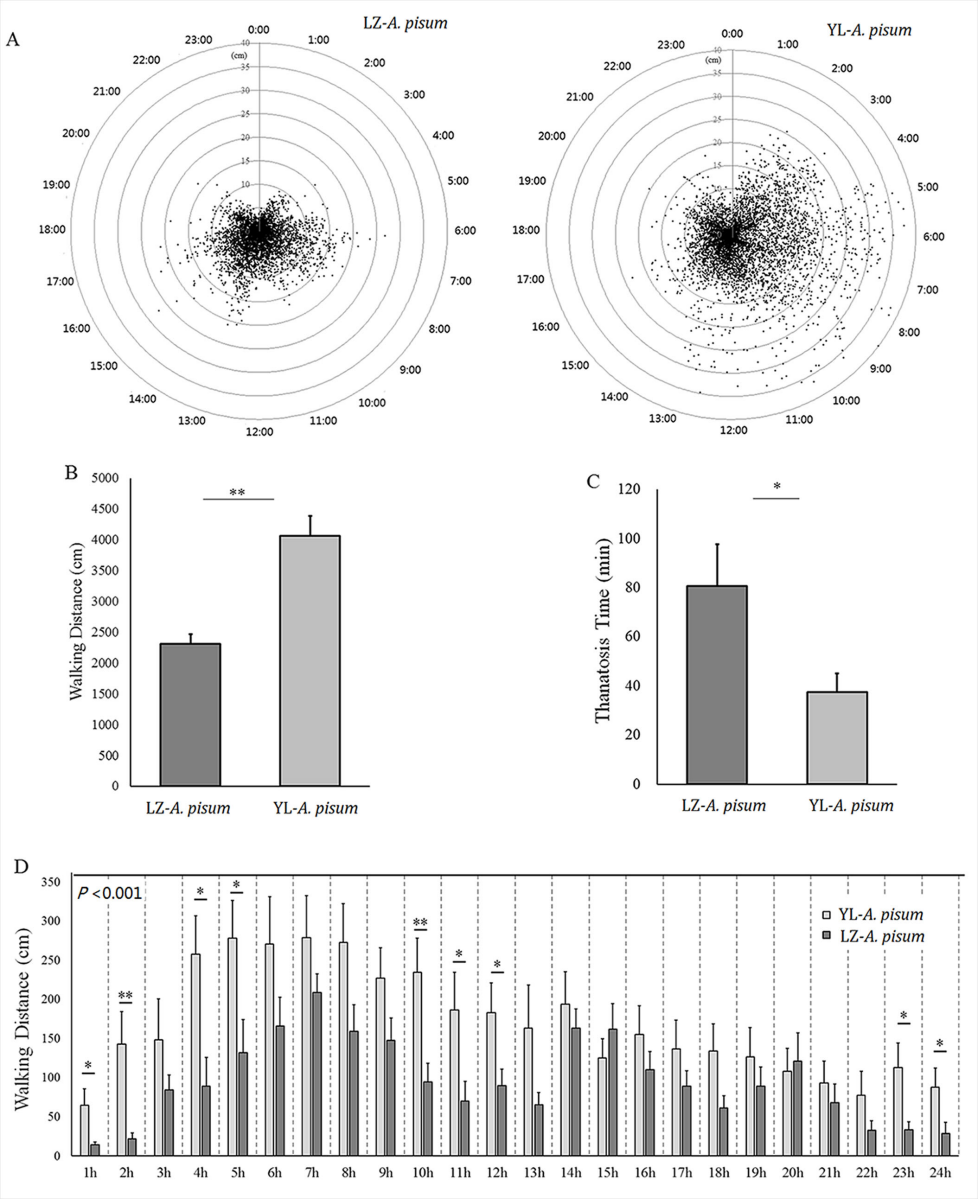
**Dispersal pattern and walking distances of the two wingless strains of *Acyrthosiphon pisum* in 24 h.** (A) The dispersal pattern and walking distances; the circle was divided hourly for 24 h, and the markers in radius represent the walking distance of each aphid in every 2 min (based on the distance to the center of the circle). (B) Total walking distances (cm). (C) Time to initiation of movement (min). (D) Total distance (mm) per hour. Values represent mean±s.e.m. from independent determinations; *P<0.05, **P<0.01 (Student's *t*-test in B and C; parameter estimates of general linear model repeated-measures in D). *P* values in D derived with repeated measures testing.

The dispersal distances of the starved aphids every 2 min are shown in Fig. 2A. The nymphs of the YL strain moved longer distances than the LZ strain. Total dispersal distance of the YL strain in 24 h was 4064.8 cm or at a speed of 169.4 cm h^−1^, as compared to 2304.6 cm or a speed of 96.0 cm h^−1^ of the LZ strain (*t*=4.867, d.f.=24, *P*<0.001) (Fig. 2B). The YL strain also started to move earlier than the LZ strain (*t*=−2.328, d.f.=22, *P*=0.029) (Fig. 2C), and was more active to walk than the LZ strain. The multivariate test of repeated-measures showed no interaction effect between time and experimental strains (*P*=0.453); and the dispersal distances were significantly related to time (*P*<0.001, tests of within-subject effects, repeated-measures of GLM). Tests of between-subject effects (repeated-measures of GLM) showed that the dispersal distances of the two aphid strains in 24 h were significantly different (*F*=23.689, d.f.=1, 24, *P*<0.001) (Fig. 2D).

### Energy reserves

**Fig. 3.**
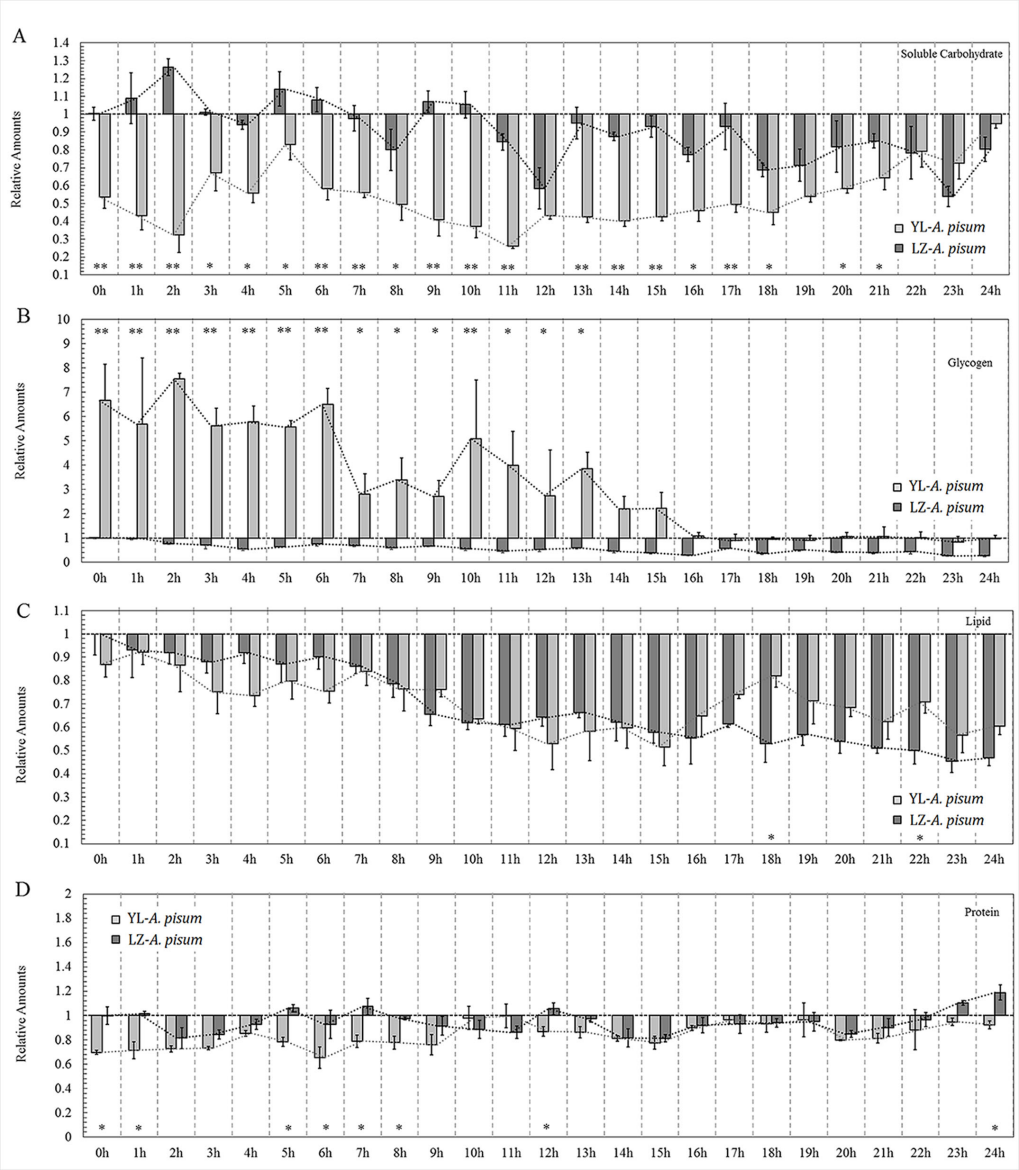
**Relative amounts of evergy reserves of the two strains of *Acyrthosiphon pisum.*** (A) Soluble carbohydrate; (B) glycogen; (C) lipid; and (D) protein. Mean value of each analyzed absolute amount in the LZ strain at 0 h was treated as 1. Values represent mean±s.e.m. from independent determinations; *P<0.05, **P<0.01 (Student's *t*-test).

Considering a strong connection between energy reserves and locomotion, we analyzed the differences of soluble carbohydrates, glycogen, proteins and lipids between the two strains. The LZ strain contained more soluble carbohydrates than the YL strain (Fig. 3A and Table S3). The soluble carbohydrate displayed a downward trend in the LZ strain, but showed an upward trend in the YL strain. The tests of between-subject effects (ANCOVA) showed strong interactions between time and experimental strains (*P*<0.001), and contrast tests showed differences at many time points between the two strains (Fig. 3A).

Based on the tests of between-subject effects (ANCOVA), interactions between time and experimental group were observed (*P*<0.001). The glycogen reserves in the earlier time points between the two strains were significantly different, and not significantly different in the later time points (Fig. 3B). The YL strain contained about sevenfold more glycogen than the LZ strain during the first 6 h, and then gradually decreased (Fig. 3B). Although both curves displayed downward trends, glycogen reserves in the YL strain declined rapidly, and almost reached the same level as in the LZ strain at the end of the experiment (Fig. 3B and Table S3).

### Proportion of located aphids

The decline patterns of the lipid reserves in the two aphid strains were similar, The tests of between-subject effects (ANCOVA) showed strong interactions between time and experimental strains (*P*<0.001), and contrast tests showed differences at 18 h between the two strains (*P*=0.003) and 22 h (*P*=0.032) (Fig. 3C and correlation test showed on Table S3).

The protein reserves also showed similar trends in quantity between the two strains. The tests of between-subject effects (ANCOVA) showed interactions between time and experimental strains (*P*=0.023), and contrast tests showed some differences between them at many time points (Fig. 3D). The amount of proteins of the two strains did not show any correlation with experimental time (*P*=0.401) (Table S3).

**Fig. 4.**
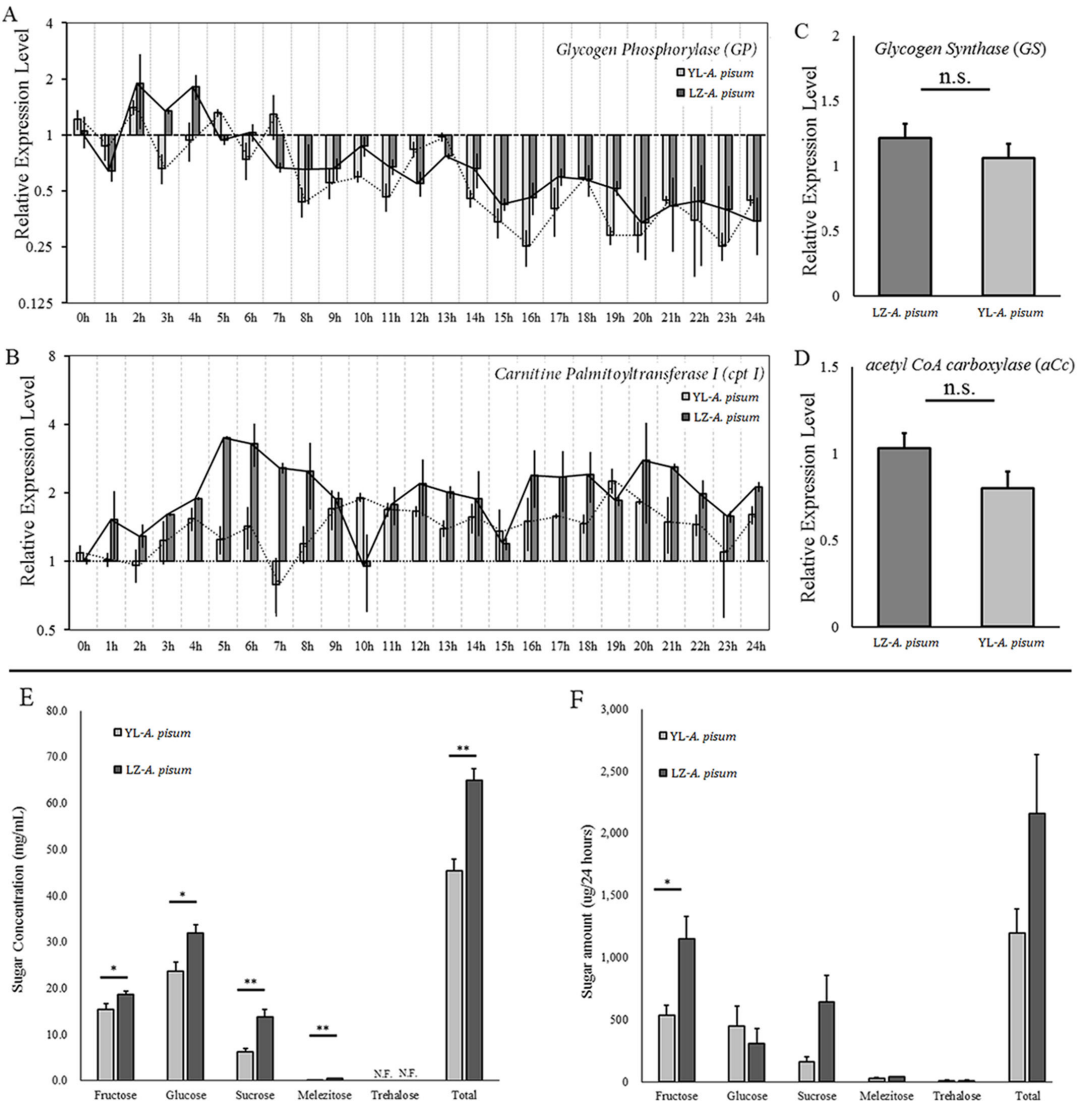
**Relative expression levels of *glycogen phosphorylase* (*GP*), c*arnitine palmitoyltransferase I* (*cpt 1*), *glycogen synthase* and *acetyl CoA carboxylase* of the two strains of *Acyrthosiphon pisum* in a 24-h starvation treatment; concentrations and amounts of the sugars in the honeydews of the two strains of *Acyrthosiphon pisum*.** (A) *Glycogen phosphorylase* (*GP*); (B) *carnitine palmitoyltransferase I* (*cpt 1*); (C) *glycogen synthase*; (D) *acetyl CoA carboxylase*. (E) Concentrations of the sugars (fructose, glucose, sucrose, melezitose and trehalose) in fresh honeydew. (F) Total amounts of sugars in the honeydews in 24 h. Values represent mean±s.e.m. from independent determinations; *P<0.05, **P<0.01 (Student's *t*-test).

The related genes in glycogen and lipid degradation pathways were analyzed. By comparing analysis of every sampling point, only 4 out of 25 sampling points were significantly different between the two strains (Fig. 4A,B and Table S4).

Tests of between-subject effects (ANCOVA) in transcription levels of *glycogen phosphorylase* (*GP*) and *carnitine palmitoyltransferase I* (*cpt 1*) showed no interactions between time and strains (*GP, P*=0.113; *cpt 1, P*=0.359). *GP* expressions of both aphid strains showed a downward trend with no significant difference between the two strains (*P*=0.113) (Fig. 4A); however the transcription levels of *cpt 1* showed an increase at the beginning and then became relatively stabilized, and the difference was significant between the two strains (*P*<0.001) (Fig. 4B).

### Glycogenesis and fatty acid synthesis

To understand what might cause the glycogen reserve differences between these two aphid strains, we analyzed a key enzyme in the glycogenesis (*glycogen synthase*, *GS*) pathway. RT-QPCR results showed no difference between the two aphid strains (*t*=1.016, d.f.=10, *P*=0.333) (Fig. 4C), indicating that the *GS* was similar between the two aphid strains in transcription level and activities.

We also analyzed the transcription difference of a key enzyme (*acetyl CoA carboxylase*, *aCc*) in fatty acid synthesis pathways between the two aphid strains, and no significant differences were obtained (*t*=1.791, d.f.=10, *P*=0.104) (Fig. 4D).

### Sugar assimilation

Considering the difficulty in sugar assimilation analysis of the two strains, we analyzed assimilation indirectly using the sugar contents of the honeydew they excreted. Five sugars were detected, including fructose, glucose, sucrose, melezitose and trehalose. The quantity of all sugars of the fresh honeydews in the LZ strain was higher than that in the YL strain (fructose: *t*=−2.356, d.f.=12, *P*=0.036; glucose: *t*=−2.849, d.f.=12, *P*=0.015; sucrose: *t*=−3.483, d.f.=12, *P*=0.005; melezitose: *t*=−6.015, d.f.=12, *P*<0.001) (Fig. 4E). The concentrations of four of the five sugars in the honeydews excreted in 24 h were not significantly different between the two aphid strains (glucose: *t*=0.702, d.f.=4, *P*=0.521; sucrose: *t*=−2.228, d.f.=4, *P*=0.090; melezitose: *t*=−2.054, d.f.=4, *P*=0.109; trehalose: *t*=0.296, d.f.=4, *P*=0.782) except for the amounts of fructose which were significant higher in the LZ strain than in the YL strain (*t*=−3.184, d.f.=4, *P*=0.033) (Fig. 4F).

### Host identification ability

**Fig. 5.**
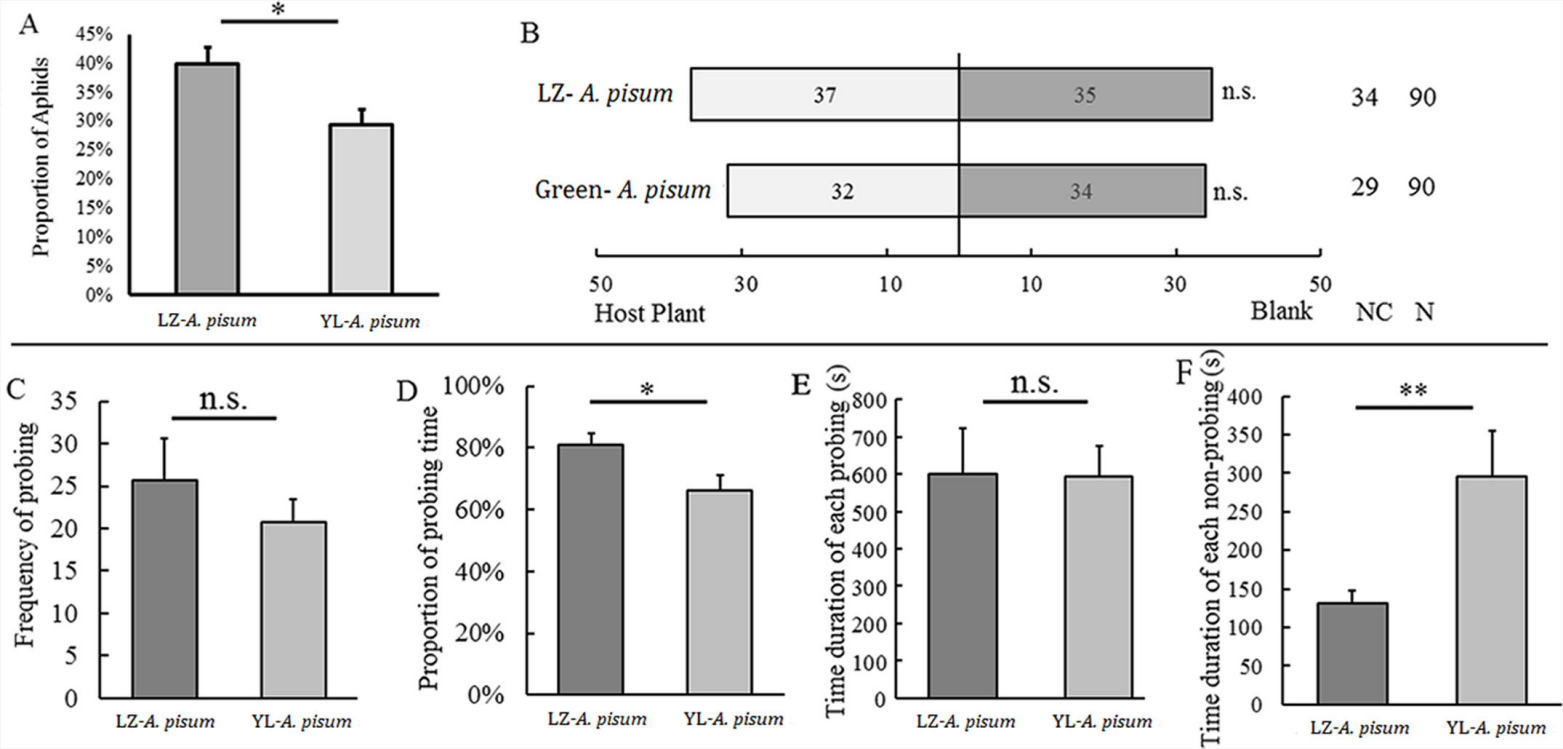
**Host leaf identification, responses to host plant volatiles and probing behaviors of the two strains of *Acyrthosiphon pisum*.** (A) Number of aphids on the leaf disc for each strain were counted; (B) Y-tube olfactometer test (NC, no choice); (C) probing frequency; (D) total probing duration; (E) duration of each probing; and (F) non-probing time. Values represent mean±s.e.m. from independent determinations; *P<0.05, **P<0.01 (Student's *t*-test).

Significantly more aphids of the LZ strain moved to the host leaf than the YL strain in 2 h (*t*=−2.828, d.f.=28, *P*=0.009) (Fig. 5A) in the petri dish arena under dark (Fig. S1B,C).

The two strains did not show different responses to plant volatiles, and neither of them could distinguish plant volatiles at the blank control (LZ: χ^2^=0.044, d.f.=1, *P*=0.833; YL: χ^2^=0.040, d.f.=1, *P*=0.842) (Fig. 5B).

### Probing behaviors

The probing frequencies were not significantly different between the two strains (26 vs 21) (*t*=0.961, d.f.=13, *P*=0.377) (Fig. 5C). However, total probing duration in the LZ strain was significantly longer than that in the YL strain (81% vs 66%) (*t*=2.248, d.f.=13, *P*=0.043) (Fig. 5D). The duration of each probing was identical (about 600 s) between the two strains (*t*=0.056, d.f.=332, *P*=0.956) (Fig. 5E), but the interval between each probing of the LZ strain was dramatically shorter than that of the YL strain (130 s vs 295 s) (*t*=−2.630, d.f.=337, *P*=0.007) (Fig. 5F).

## DISCUSSION

Our results confirmed that there were significant differences between the two strains of apterous *A. pisum* in host relocation. Some biological differences were observed between the two aphid strains. These two *A. pisum* strains were identified by COI sequences (primers shows in Table S1) and confirmed as the same species. Our preliminary tests also showed that the two *A. pisum* strains could mate and produce normally. The LZ strain was superior at host seeking and leaf identification under starvation, but the movement ability of the YL strain was actually stronger than the LZ strain. Considering the relationship between energy reserves and movement ability of the two aphid strains, this phenomenon was probably caused by higher glycogen reserves that provide more energy for walking a longer distance and spreading out a wider range. We found that the key enzyme activities between the two strains were not significantly different, and the differences in glycogen reserves between the two strains might be due to the differences in sugar assimilation. On one hand, the YL strain took in more sugars as the substrates for glycogenesis than the LZ strain. On the other hand, the pre-feeding behaviors (probing) between the two strains were different, and the high probing frequency of the LZ strain might help the aphids to quickly and accurately identify their host.

Although wingless aphids mostly are sedentary and can only walk a relatively short distance, their dispersal behaviors are also important for aphids population (Braendle et al., 2006; Brisson, 2010; Irwin et al., 2007), and this has been confirmed by several studies (Phelan et al., 1976; Alyokhin and Sewell, 2003; Kunert et al., 2010; Ben-Ari et al., 2015). Comparing with fully developed winged aphids, wingless aphids have degenerations in some of their sense organs and lose locomotion abilities, resulting in different dispersal ranges (Braendle et al., 2006; Bromley et al., 1980; Kring, 1977; Moran, 1992; Park and Hardie, 2002). The wingless nymphs of the two pea aphid strains showed different host seeking and movement abilities, and the movement ability of the LZ strain was weaker than the YL strain; also, the time to initiation of movement time was longer, which could be caused by having a lower glycogen reserve. However, the LZ strain had a higher probing frequency, which could be a compensation for them to cope with their low glycogen reserves. In contrast, the YL strain had a high level of glycogen reserve, and correspondingly had stronger locomotion ability than the LZ strain. Based on our studies, the sugar assimilation difference could be the underlying cause, and it could lead to differing glycogenesis and stamina levels in dispersal behaviors and force aphids to evolve two strategies distantly; however, the bigger and heavier strain, which is LZ, was observed in lower glycogen reserving. We believe that there is no certain connection between body weight (and body length) and glycogen reserve, as the smaller aphids survived longer and showed greater locomotion ability; however considering their biological and behavioral differences, the mechanism behind this might be complicated.

The fluctuation and variation of numbers of aphids seeking host plant leaves were well correlated with their locomotion patterns in both aphid strains (Fig. 1D and Fig. 2D), indicating that host-seeking patterns of the aphids could be determined by their own locomotion abilities. The great variation in locomotion in the LZ strain may be related to body-color shifting (Fig. S3), especially under starvation, as their body color and moving behavior could be affected by deteriorated nutrition (Tabadkani et al., 2013; Valmalette et al., 2012); however the connection between escaping strategies and body color is unknown and more evidence is needed.

Other biological characteristics of the wingless *A. pisum* could also modify their host seeking strategies. It had already been proven that wingless aphids have degeneration in their compound eyes, ocelli, and antennae (less rhinaria) (Braendle et al., 2006, Bromley et al., 1980; Kring, 1977; Moran, 1992; Park and Hardie, 2002). Our studies showed that both aphid strains had no response to host plant volatiles, and this could be due to antennae degeneration. Previous studies found that plant volatiles are less important in host finding in some aphid species (Brady, 1997; Gish and Inbar, 2006; Döring, 2014); nonetheless, the probing behavior seems important in host leaf identification, and the higher probing frequency of the LZ strain could save their energy in host location. It is reported that feeding behaviors are mostly controlled by the nervous system, and some neurotransmitters, such as octopamine and some neuropeptides, may play vital roles in insect's host seeking processes (Long and Murdock, 1983; Vehovszky et al., 1998; Cohen et al., 2002). Other biological indicators, such as survival duration under starvation, could strongly affect an insect's dispersal range. Based on our results, the YL strain survived longer under starvation than the LZ strain, which enable the YL strain to survive longer, walk for a longer time, and disperse over a wider range, and have an opportunity to find new plants as compared with the LZ strain (Fig. S2).

The different strategies of the two wingless strains for short distance movement could give them different ecological advantages. The LZ strain could effectively find a host without using too much energy as they have a relatively high probing frequency; however, this strategy could limit their dispersal range. The YL strain showed a different strategy, reserving much more glycogen and low probing frequency as compared with the LZ strain, so that they could tolerate longer period of starvation, move longer distances and disperse over wider ranges. Although there could be a chance that the original host plant is in a good enough quality and quantity for the aphids to stay and do not need to walk away and waste their energy to find a new food resource, it would be an advantage for the aphids if they are able to quickly walk away from the original location and establish a new colony. This could be also helpful to reduce competition for descendants of the aphids (Fig. S5).

Although only two strains of *A. pisum* were tested, they showed two different strategies for same threat. We assumed that natural selection of the aphids might cause differences in response between the two strains to the same stress environments. However, at present, we still do not know which environmental cues are responsible for these adaptations in aphids, and how the aphids cope with these pressures while changing their biological characteristics. More studies are needed to explain the differences in physical and biological mechanisms of sugar assimilation and probing behaviors between these two strains of *A. pisum,* and more strains of *A. pisum* need to be tested for a universal explanation. The possible connection between the aphids' locomotion and body-color of the two strains *A. pisum* should also be further investigated.

## MATERIAL AND METHODS

### Experimental insects and plants

Two strains of *A. pisum* were observed in different escaping strategies: one strain, which was red, was collected in *Medicago sativa* from Lanzhou, Gansu Province, China, 2014 (marked LZ strain); and another strain, which was green, was obtained from our laboratory colony in Yangling, Shaanxi, China (marked YL strain, originally collected in *Vicia faba* from New York, USA, 2009). Both strains were cultured on broad bean (*V. faba* L., var. ‘Jinnong’) under long-day conditions (16 h light:8 h dark; 20±1°C) for more than 30 generations at the Key Laboratory of Applied Entomology, Northwest A&F University, Yangling, Shaanxi, China. All aphids were reared at a relatively low density (less than 30 individuals per plant) for more than three generations before they were used in all experiments.

### Experiments design

#### Biological differences of two pea aphid strains

Body length and weight of two pea aphid strains were measured (the lengths of antenna and cornicle were not included). Twelve hours after molting, the aphids of each stage were collected and body length were measured using Zeiss SteREO Discovery V12 stereomicroscope (with Plan ApoS 2.3X FWD 10 mm and AxioCam MRc5; light source: KL1500LCD, Zeiss, Germany). We measured 59 individuals of each aphid stage. Adult leg length (tibia of the third pair of legs) of two strains were also measured by same protocol.

Twelve hours after molting, the fourth instar nymphs and adult aphids were collected and weighed accurately using a high precision electronic balance (LL3000, Thermo, USA) at room temperature. At least 50 individuals of each stage were weighed.

#### Host location

To compare how fast the two wingless strains found food, we designed an experimental device which made it relatively difficult for the aphids to find a plant leaf disk in an arena (Fig. S1A). Only fourth instar nymphs (12 h after molting) were used because the adults could produce nymphs during the experiment, which may affect the movement and searching behavior. Fifty individuals from each strain were placed in the right container of the arena. The presence of aphids on the leaf disk in the arena was recorded using a digital camera (Lumix GH2, Panasonic, Matsushita Electric Industrial Co., Ltd, Ōsaka, Japan) through an observation window for 24 h (Fig. S1A). Numbers of aphids that found and fed on the leaf disk were counted every 30 min. Ten replications of each aphid strain were tested.

#### Locomotion under starvation

Fourth instar nymphs (12 h after molting) from each strain were used in this experiment. First, an aphid was placed in a petri dish (φ 30 mm) without diet, and the locomotion of the aphid was recorded for 24 h using the same camera as described above. Each aphid was only monitored once, and 13 aphids (replications) were tested in each strain. The walking distance data were collected every two minutes. Locomotion data were collected by EthoVision XT (Noldus Information Technology, Wageningen, The Netherlands). The video was replayed, and the walking distance and locomotion data were analyzed using EthoVision XT software (Noldus Information Technology, Wageningen, The Netherlands).

#### Biochemical and molecular analysis

Fourth instar nymphs (12 h after molting) of the two wingless strains were placed in transparent plastic dishes (90 mm in diameter; 20 individuals per dish) for 24 h. The aphids were collected every hour with three repetitions. The aphids were freeze-dried for 36 h in a lyophilizer (Heto PowerDry LL3000 Freeze Dryer, Thermo Fisher Scientific, USA), and weighed using the same high precision electronic balance as described above at ambient temperature. The aphids were transferred into a 1.5 ml microtube, homogenized by a micropestle, and dissolved in 800 μl solution buffer (100 mM KH_2_PO_4_, 1 mM DTT, 1 mM EDTA, pH 7.4) for further analysis.

##### Glycogen and soluble carbohydrate assays

After all samples were dissolved in the solution buffer mentioned above, each tube with the aphid samples was centrifuged at 20,000 ***g*** for 15 min at 4°C to remove deposition from the mixture. The supernatant was then transferred into a new tube for soluble carbohydrate assay; and the deposition was washed twice using methanol for glycogen assay. Both glycogen and the soluble carbohydrates were measured using the colorimetric method based on enthrone reagent and glucose as the standard as described in Foray et al. (2012).

##### Protein assay

After all samples were dissolved in the solution buffer, each tube with the samples was centrifuged at 180 ***g*** for 15 min at 4°C, and the protein content mixture was measured using a modified Bradford protein assay kit (C503041-1000, Sangon Biotech, Shanghai, China).

##### Lipid assay

After all samples were dissolved in the solution buffer, 160 μl NaOH (6 N) was added for fat hydrolysis in each tube, and was water-bathed (75°C) for 3 h. The hydrolytic fatty acid content mixture was measured using a non-esterified free fatty acids (NEFA) assay kit (A042, Nanjing Jiancheng Bioengineering Institute, Nanjing, China).

### Transcription analysis

To analyze reaction difference in glycogenesis, glycogenolysis and lipid synthesis and degradation (beta oxidation) between the LZ and YL strains, we picked four rate-limiting genes (glycogen synthase, glycogen phosphorylase, acetyl CoA carboxylase and carnitine palmitoyltransferase I for glycogenesis, glycogenolysis, lipid synthesis, and lipid degradation respectively) in these metabolic processes, and their transcription levels were detected. Fourth instar nymphs (12 h old) of the wingless YL and LZ strains were separately placed into transparent plastic dishes (φ 90 mm), 20 individuals per dish. Each test had three repetitions. The samples were then used for transcription analysis.

The aphids were quick-frozen using liquid nitrogen immediately after collection. RNA was extracted with RNAiso Plus (Takara, Japan), and cDNA was synthesized using a PrimeScript™ RT reagent kit with gDNA Eraser (Takara, Japan). Quantitative real-time PCR (qRT-PCR) was performed with SYBR^®^ Premix Ex Taq™ II (Takara, Japan) in an IQ-5 system (Bio-Rad, USA). The primers were designed by Primer-BLAST of NCBI online (http://www.ncbi.nlm.nih.gov/tools/primer-blast/index.cgi?LINK_LOC=BlastHome) (Table S1).

### Sugar assimilation

In order to further understand what causes the differences in energy reserves between the two strains, sugar assimilation was determined by detecting sugar concentration in the honeydew. Honeydew droplets were collected as described by Völkl et al. (1999) and Fischer et al. (2005) using a microcapillary (P-97 Micropipette Puller; Sutter, CA, USA; pulling program: Pull=100, VEL=100, Time=100). Ten wingless aphids (fourth instar nymphs) were reared in a petri dish (30 mm in diameter) with a leaf of *V. faba*, and a thin layer of 1% agar was placed in the dish bottom to keep the leaf fresh. Ten dishes were prepared for honeydew collection. Freshly collected honeydew droplets were diluted 100 times using ddH_2_O for further analysis. To analyze sugar excretion in 24 h, an aphid was placed in a petri dish (φ 30 mm), and all honeydew droplets excreted by the aphid were collected and dissolved in 300 μl ddH_2_O for further analysis. The diluted honeydew samples were analyzed using LTQ XL linear ion trap mass spectrometer (Thermo Scientific, Waltham, MA, USA). The sugars were scanned and fragmented using data dependent MS/MS. Masses of precursor and product ions and collision energy for each sugar were shown in Table S2. The data were acquired and processed using Xcalibur 2.1 software (Thermo Fisher Scientific, Waltham, MA, USA). Quantification was achieved by external standard sugar mixtures of known concentrations.

### Host leaf identification

Fourth instar nymphs were starved for 12 h before they were used in all experiments. First, we attempted to determine how fast the aphids identify a host leaf. The aphids were placed in a petri dish (150 mm in diameter) filled with 1% agar. A host leaf disc (30 mm in diameter) was placed in one side in the dish, and 50 aphids were placed 55 mm away from the leaf-disc in another side as shown in Fig. S1B and C. The experiment was conducted in the dark, and number of aphids moved to the leaf disc was counted 2 h after the aphids were introduced. The experiment was replicated 15 times.

Second, we used a Y-tube olfactometer to test the responses of adult *A. pisum* to their host plant, *V. faba,* and clean air (control) (Bailey et al., 1995). The Y-tube had an internal diameter of 0.6 cm, and the two arms were connected to two flow meters and glass vessels (2.5 liters) containing odor sources. A 2-week old *V. faba* plant was prepared in one side, and a pot with media without a plant was putted in another. Air was filtered by activated charcoal and pushed through each of the Y-tube arm at 0.20 liters/min. We considered the aphid had made a choice when it crossed an arm for 5 cm distance and stayed there for more than 30 s within a 5-min period, or otherwise as ‘no choice’. The arms were interchanged after 5 aphids were tested, and the odor sources were interchanged every 10 aphids. A new clean Y-tube glass was used after every 30 aphids. After each experiment, all parts of the whole olfactometer were washed with ethanol and dried at 60°C. The experiment was also carried out in a dark room with three 20 W fluorescent lamps on the top of the Y-tube.

### Probing behaviors on a non-host plant

After we found that host volatiles did not affect host-plant location between the two strains, we undertook this experiment to determine their feeding behaviors. We used a non-host plant (wheat, *Triticum aestivum* var. ‘Xinong 979’) for the aphids to stimulate the non-host natural situation. It was been designed for comparing their probing behaviors, an electrical penetration graph system (EPG) was used for measuring 24 h after they were starved for 12 h, the protocol was followed as described by Garzo et al. (2015) and Sarria et al. (2009). The EPG signals were acquired and analyzed using Stylet+software (1.0.0.0, EPG-Systems, Wageningen, The Netherlands). Only probing and non-probing times were analyzed because some probing waveforms were not identifiable because wheat was not the host of *A. pisum*.

### Statistical analysis

All experimental data were subjected to statistical analysis using Student's *t*-test, univariate repeated measures (General Linear Model), ANCOVA or Chi-square test using SPSS (version 22; SPSS Inc., Chicago, IL, USA). The locomotion data were analyzed using Noldus EthoVision^®^ XT (version 9.0; Noldus Information Technology b.v., Wageningen, The Netherlands).
